# Effects of short-term nitrogen and phosphorus addition on soil bacterial community of different halophytes

**DOI:** 10.1128/msphere.00226-24

**Published:** 2024-04-29

**Authors:** Zehao Zhang, Lijie Wang, Tian Li, Zhanyong Fu, Jingkuan Sun, Rui Hu, Yao Zhang

**Affiliations:** 1 Shandong Key Laboratory of Eco-Environmental Science for Yellow River Delta, Shandong University of Aeronautics, Binzhou, China; 2 College of Forestry, Shandong Agricultural University, Taian, China; University of Wisconsin-Madison, Madison, Wisconsin, USA

**Keywords:** nitrogen and phosphorus addition, bacterial community, halophytes, rhizosphere soil, Yellow River Delta

## Abstract

Soil microbial community composition and diversity are often affected by nutrient enrichment, which may influence soil microbes to affect nutrient cycling and plant community structure. However, the response of soil bacteria to nitrogen (N) and phosphorus (P) addition and whether it is influenced by plants remains unclear. By 16S rRNA sequencing, we investigated the response of the rhizosphere and bulk soil bacterial communities of different halophytes (salt-rejecting, salt-absorbing, and salt-secreting plant) in the Yellow River Delta to short-term N and P addition. The response of rhizosphere bacterial diversity to N and P addition was opposite in *Phragmites communis* and *Suaeda salsa*. N addition increased the rhizosphere soil bacterial α-diversity of *S. salsa* and *Aeluropus sinensis*, while P addition decreased the rhizosphere bacterial α-diversity bacteria of *S. salsa*. The N and P addition had a weak effect on the rhizosphere bacterial community composition and a significant effect on the bulk soil bacterial community composition of halophytes. The *S. salsa* and *P. communis* bulk soil bacterial community were mainly influenced by P addition, while it was influenced by N addition in *A. sinensis*. N and P addition reduced the difference in bacterial community composition between the two types of soil. N and P addition increased the eutrophic taxa (*Proteobacteria* and *Bacteroidetes*) and decreased the oligotrophic taxa (*Acidobacteria*). Redundancy analysis showed that soil organic matter, salt, and total N content had significant effects on the bacterial community composition. The results clarify that the response of soil bacterial communities to N and P additions is inconsistent across the three halophyte soils, and the effect of plant species on the bacterial community was stronger than short-term N and P addition.

**IMPORTANCE:**

The bulk soil bacterial community was more affected by nutrient addition. Nitrogen (N) and phosphorus (P) have different effects on bacterial community. Soil organic matter is a key factor influencing the response of bacterial community to nutrient addition. N and P influence on bacterial community changes with plants.

## INTRODUCTION

Since the 20th century, human activities such as fossil fuel burning and the use of nitrogen (N) and phosphorus (P) fertilizers have significantly impacted the biogeochemical cycles of N and P (1). In China’s natural ecosystem, the input of N to the soil has increased from 15.3 Tg year^−1^ to 175–259 Tg year^−1^ between 1980 and 2011, while the input of P has increased from 0.3 Tg year^−1^ to 14–16 Tg year^−1^ during the same period (2). The enrichment of N and P in soil can have significant impacts on ecosystem stability. Soil microorganisms play a crucial role in terrestrial ecosystems by contributing to essential biogeochemical processes and activities within the soil (3–5). Environmental changes can have an impact on the community composition and structure of soil microorganisms, which in turn can affect the nutrient cycling process and ultimately the growth of plants. Despite this, the exact impact of environmental change on soil microorganisms is still not fully understood. The study of soil microbial community variation in response to N and P fertility is crucial for understanding how global change and human activity affect nutrient cycling in subsurface ecosystems.

The effect of N addition on the composition of microbial communities has been extensively studied. Zeng et al. (6) found that N addition could directly affect the bacterial community composition of the temperate steppe ecosystem. Some studies have found that N input influences the soil bacterial community indirectly through changes in other soil factors (e.g., N effectiveness, pH) or plants (e.g., plant diversity and changes in plant carbon sources). Additionally, some studies have revealed that N input can indirectly influence the soil bacterial community by altering other soil factors such as N effectiveness and pH or through changes in plant diversity and carbon sources (6–8). It has been shown that the effect of N application on the soil microbial community structure and diversity is not uniform (9), which indicates the complexity of the effect of N addition on the microbial community, a process that depends on ecosystem type and soil properties as well as the type of N addition, dose, and time duration of addition (10–12). For example, N addition reduced bacterial community richness in forest soil, while it had no effect on bacterial community in grassland ecosystems (13).

Compared to N addition, a few studies have focused on the effect of P addition on soil microbial diversity and microbial community structure. According to Ma et al. (14), the injection of P altered the bacterial community structure of primary and secondary forests directly and indirectly through changing soil environmental factors. Ling et al. (15) found that changes in available phosphorus (AP) content caused by P addition were a key factor in altering the diversity and composition of soil bacteria. While Huang et al. (16) found that P addition changed the bacterial community composition by changing soil pH. Furthermore, P addition has also been found to have less impact on the composition and structure of microbial communities compared to N addition (17, 18). These results suggest that the effect of P addition on soil microbial community is inconsistent and may be related to local environmental factors.

Although the effects of N and P addition on bacterial communities have been extensively reported, these studies focus on bacterial community change under fertilizer application methods and applied dose conditions. It is unclear whether there is a correlation between the response of soil microorganisms and plants to N and P addition. Indeed, the impact of vegetation on the composition and diversity of soil bacterial communities is substantial (19). For example, when the plant is deficient in P content, the root system indirectly affects the bacterial community by secreting organic acids to activate the active P. On the other hand, N and P addition changes the shape of the root (diameter, surface area, biomass, etc.) affecting the bacterial community (20). However, these previous studies focused on the effect of N and P addition on the soil microbial community, ignoring the fact that the plant-soil-microbial community should be an integral system. Therefore, the rhizosphere and bulk soil of three typical halophytes (*Suaeda salsa*, salt-absorbing plant; *Phragmites communis*, salt-rejecting plant; and *Aeluropus sinensis*, salt-secreting plant) selected in the Yellow River Delta (YRD) to investigate the effects of plants and N and P addition on the soil bacterial community by 16S rRNA sequencing. We predict that plant and N and P addition can affect bacterial communities, and plant effects on bacterial communities are greater than N and P addition.

## MATERIALS AND METHODS

### Site description

The study region was in the YRD, Shandong Province, China (118°44'2"E, 38°1'18"N). The study region has a temperate semi-humid continental monsoon climate with 550–640 mm of precipitation per year and an average annual temperature of 12.6°C. The N deposition in the growing season of the study area is 2.26 g m^−2^, and the content of AP is 4.04–6.28 mg kg^−1^. Salinity is a major limiting factor for plant growth. The average soil salinity of this site is 4.48‰. The vegetation is mainly composed of halophytes: *P. communis*, *A. sinensis*, *S. salsa*, and *Tamarix chinensis*.

### Experiment design

Three single halophyte distribution areas were selected. The plants in each distribution area are *P. communis*, *A. sinensis*, and *S. salsa*, respectively. Four treatments were set up, namely, control (CK, no addition), N addition (N, 15 g/m^2^), P addition (10 g/m^2^), and N-P co-addition (NP, 15 g/m^2^ N, 10 g/m^2^ P) (2). Twelve 1 × 1 m sample squares were set for each plant distribution area. Three parallel experiments were designed for each treatment. N addition treatment was randomly conducted using urea. P addition treatment was conducted using KH_2_PO_4_, and an equal volume of pure water was added for CK treatment.

### Soil sampling

Three months following the N and P addition, in July 2021, sample collection was completed. Soil adjacent to the root system was considered rhizosphere soil. The soil at 15 cm depth on the ground without plant growth in the sample square was considered bulk soil. Seventy-two soil samples were collected. Each soil sample was divided into two parts, one for soil environmental factor measurement and the other for 16S rRNA analysis.

### Measurements of soil physical and chemical properties

The potassium dichromate volumetric was used to measure the soil organic matter (SOM) content. The elemental analyzer (Vario EL III, Elementar, Germany) was used to measure the total N (TN) and total C (TC) content. pH meter and conductivity meter were used to measure the pH and soil salt, respectively. The content of AP was quantified using extraction-molybdenum antimony anti-colorimetry. Molybdenum blue method was used to measure the content of total phosphorus (TP).

### Extraction, sequencing, and analysis of soil DNA

Genomic DNA was extracted using the CTAB method. Soil samples were treated with 1,000 µL CTAB lysis buffer containing lysozyme at 65°C, with periodic inversion for complete sample disruption. The supernatant was collected after centrifugation and subjected to a phenol (pH 8.0):chloroform:isoamyl alcohol (25:24:1) extraction, followed by a second chloroform:isoamyl alcohol (24:1) extraction. The resulting supernatant was precipitated with isopropanol, washed with 75% ethanol, air-dried, and dissolved in ddH_2_O. Subsequent digestion of RNA was performed by adding 1 µL RNase A at 37°C for 15 minutes. The 1% agarose gel electrophoresis was used to detect the DNA purity and concentration.

Using specific primers 341F and 806R, the V3–V4 region of the bacterial 16S rRNA gene was amplified by a specific primer. PCR conditions include an initial denaturation step at 98°C for 1 min, followed by 30 cycles comprising denaturation at 98°C for 10 s, annealing at 50°C for 30 s, and extension at 72°C for 30 s. The reaction concludes with a final extension step at 72°C for 5 min. All PCRs were carried out with 15 µL of Phusion High-Fidelity PCR Master Mix (New England Biolabs).

The products were purified using the AxyPrep DNA Gel Extraction Kit (Qiagen, Germany) after being detected by 2% agarose gel electrophoresis. High-throughput sequencing was conducted using the MiSeqPE300 platform from the Illumina corporation (Wekemo Tech Group Co., Ltd. Shenzhen, China).

The raw data FASTQ files were processed by Qiime2 (2019.1). The original sequences were subjected to quality control, trimming, denoising, and merging using the DADA2 plugin in Qiime 2. Following the removal of chimeric sequences, a final feature sequence table was obtained. Using the QIIME2 feature-classifier plugin, the representative sequences of ASVs were aligned to the pre-trained GREENGENES database (13_8) with a 99% similarity threshold, resulting in a table containing taxonomic classification information for the identified species. Diversity calculation using the QIIME2 core-diversity plugin.

### Statistical analysis

The Duncan test was used to compare the differences between the treatments. Redundancy analysis (RDA) was used to examine the connection between soil bacterial communities and soil physicochemical characteristics. The differences in bacterial community composition between the treatments were analyzed by principal coordinate analysis (PCoA). PERMANOVA and ANOSIM were used to evaluate the effects of N addition, P addition, and N-P interaction on the soil bacterial community composition. To analyze the association between soil characteristics and bacterial diversity, linear regression analysis was used. R software (version 4.1.0; http://www.r-project.org) was used for data analysis. The agricolae (1.3–5) package was used for the Duncan test (21). RDA, PCoA, PERMANOVA, and ANOSIM were completed using the vegan (2.6–2) package (22). The figure display and linear regression analysis were running by ggplot2 (3.3.6) and ggpubr (0.4.0) package (23, 24).

## RESULTS

### Soil bacterial community α-diversity

**Fig 1:**
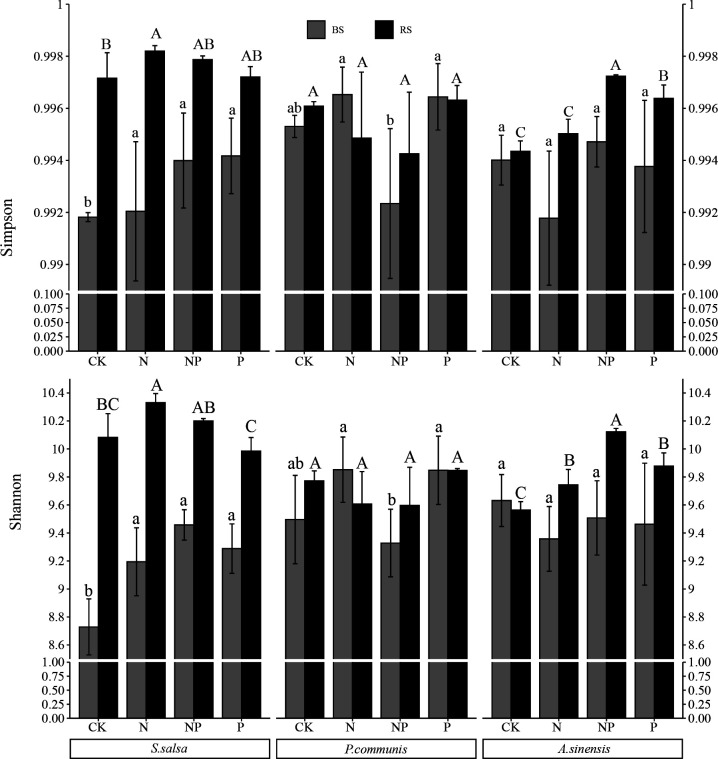
The α-diversity of the soil bacterial community in halophytes at different treatments. Different uppercase and lowercase letters indicate that the differences between treatments are significant in bacterial community diversity of rhizosphere and bulk soils, respectively. The error bars indicated SD. BS, bulk soil. RS, rhizosphere soil.

With the increase in the sequence number (Fig. S1), the increase in species richness gradually decreases and then flattens out, indicating that the sampling is reasonable, and therefore, the 72 soil samples are sufficiently rich to reflect the real situation of soil microbial communities. Two-way analysis of variance (ANOVA) (Table 1) showed that N and P addition had a significant effect on the bacterial diversity of *S. salsa* bulk soil and *A. sinensis* rhizosphere soil. The bacterial community Shannon index in *S. salsa* rhizosphere soil and *P. communis* bulk soil was significantly affected by N addition and interaction of N and P addition, respectively. N and P addition as well as the interaction of N and P addition had no significant effect on the bacterial diversity of *P. communis* rhizosphere soil and *A. sinensis* bulk soil. The rhizosphere soil bacterial community α-diversity responded differently to N and P addition in different plants (Fig. 1). In the *S. salsa*, the bacterial community α-diversity of rhizosphere soil was significantly higher than that of bulk soil. Compared to the CK treatment, the bulk soil bacterial α-diversity was significantly higher in both N and P addition as well as co-addition. The N addition treatment had a significantly increasing effect on the rhizosphere soil Shannon index bacteria in *S. salsa*, while the P addition had a significantly decreasing effect on it. In *A. sinensis*, the bacterial α-diversity in bulk soil was higher in CK treatment than it was in rhizosphere soil. While under other treatments, the opposite was observed. N and P addition as well as co-addition treatment significantly increased bacterial diversity in the rhizosphere soil of *A. sinensis* (*P* < 0.05). In *P. communis*, soil bacterial diversity in CK and NP addition treatment showed greater diversity in rhizosphere soil than in bulk soil. N and NP addition treatment reduced rhizosphere soil bacterial diversity.

**TABLE 1:** Two-way ANOVA of Shannon index of bacterial community^*a*^

Species	Factor	Rhizosphere soil			Bulk soil		
		DF	F	*P*	DF	F	*P*
*S. salsa*	N	1	**15.040**	**0.010**	1	**8.509**	**0.050**
	P	1	3.662	0.092	1	**14.318**	**0.050**
	N*P	1	0.075	0.791	1	1.857	0.210
*P. communis*	N	1	3.898	0.084	1	0.294	0.602
	P	1	0.095	0.766	1	0.328	0.583
	N*P	1	0.168	0.693	1	**8.464**	**0.050**
*A. sinensis*	N	1	**21.754**	**0.010**	1	0.452	0.520
	P	1	**57.537**	**0.001**	1	0.003	0.958
	N*P	1	0.508	0.496	1	0.876	0.377

^*a*^ DF, degrees of freedom. Boldface indicates that the *P*-value is less than 0.05.

### Principal coordinate analyses

**Fig 2:**
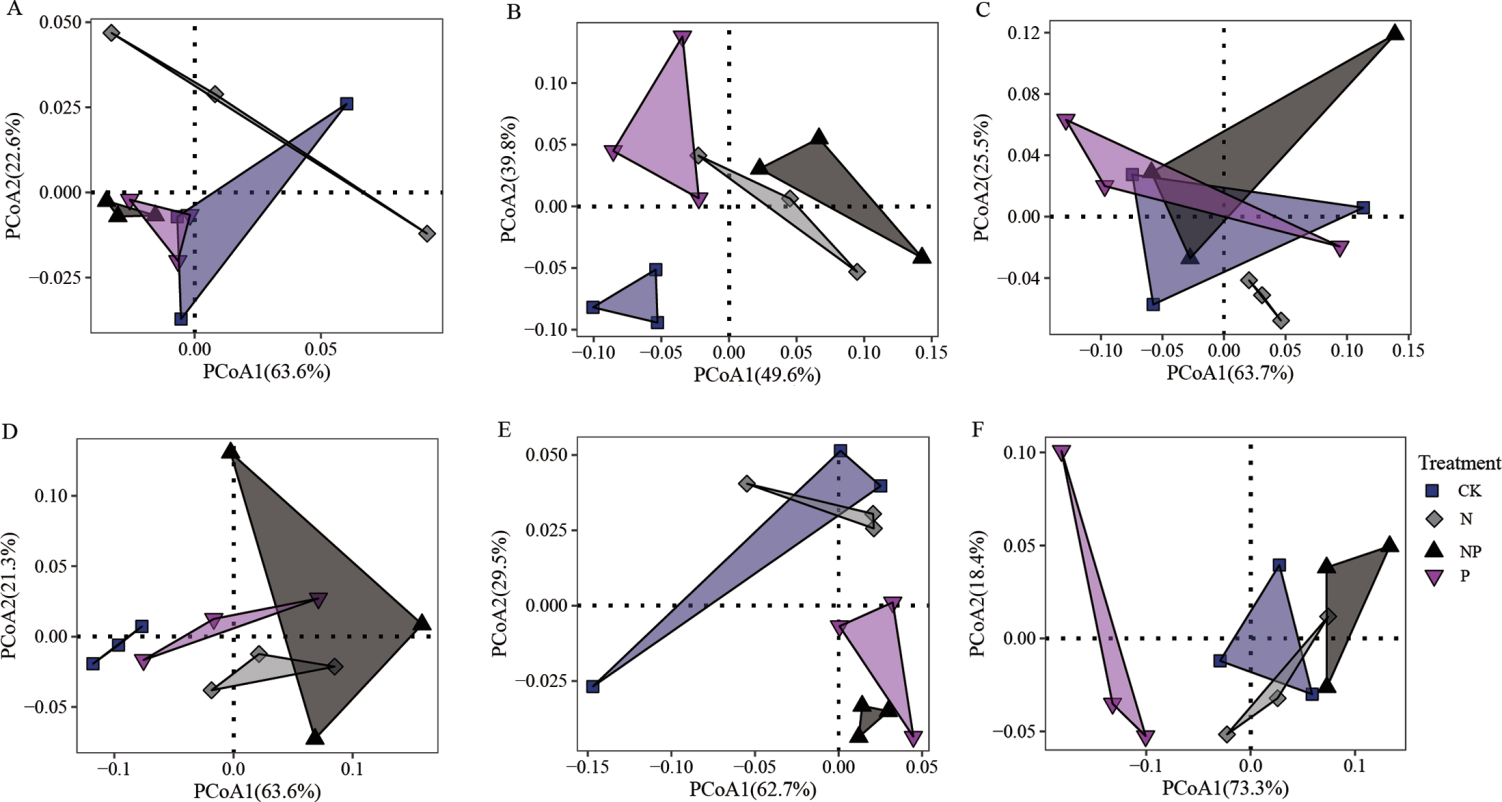
PCoA of the soil bacterial community of different halophytes at different N and P addition treatments. (A) *S. salsa* rhizosphere soil. (B) *S. salsa* bulk soil. (C) *P. communis* rhizosphere soil. (D) *P. communis* bulk soil. (E) *A. sinensis* rhizosphere soil. (F) *A. sinensis* bulk soil.

PCoAs showed that the first two axes have a better interpretation of the top 10 phyla of the relative abundance of the bacterial community (Fig. 2). Nutrient addition treatment had a greater effect on the bacterial community structure of rhizosphere soil than that of bulk soil. The addition of N and P has an insignificant effect on the rhizosphere soil of *S. salsa* and *P. communis* (Fig. 2A and C). The distance between the P and co-addition treatment and the CK and N addition treatment in the rhizosphere soil of *A. sinensis* indicated that the P addition changed the community structure of bacteria (Fig. 2E). PERMANOVA (Table S1) showed that *P* had a significant effect on the rhizosphere bacterial community structure of *A. sinensis*. The structure of the bulk soil bacterial community of all three halophytes was altered under N and P addition. The structure of the bulk soil bacterial community of *S. salsa* was significantly affected by N and P addition (Fig. 2B), the structure of the bulk soil bacterial community of *P. communis* was affected by N addition (Fig. 2D), and the bulk soil bacterial community of *A. sinensis* was mainly affected by both N and co-addition (Fig. 2F). In addition, in the whole data, the PERMANOVA test (Table 2) indicated that plant had the greatest influence on bacterial community structure (*R*^2^ = 0.247, *P* < 0.001), followed by soil (*R*^2^ = 0.132, *P* < 0.001), plant-soil interaction (*R*^2^ = 0.065, *P* < 0.001), N addition (*R*^2^ = 0.057, *P* < 0.001), soil-N addition interaction (*R*^2^ = 0.031, *P* < 0.01), plant-soil-P addition interaction (*R*^2^ = 0.027, *P* < 0.05), P addition (*R*^2^ = 0.022, *P* < 0.05), and N-P addition interaction (*R*^2^ = 0.016, *P* < 0.05), respectively. The interactions of soil and N addition were significant, which indicated that the effects of N addition were different for soil position.

**TABLE 2:** PERMANOVA (per = 999) of variance for bacterial community structure using plant species, soil type, N addition level, P addition level, and interactions as fixed effects^*a*^

Factor	DF	*R* ^2^	*P*
Plant	2	**0.247**	**0.001**
Soil	1	**0.132**	**0.001**
N	1	**0.057**	**0.001**
P	1	**0.022**	**0.011**
Plant*soil	2	**0.065**	**0.001**
Plant*N	2	0.020	0.109
Soil*N	1	**0.031**	**0.002**
Plant*P	2	0.023	0.064
Soil*P	1	0.010	0.163
N*P	1	**0.016**	**0.03**
Plant*soil*N	2	0.016	0.25
Plant*soil*P	2	**0.027**	**0.033**
Plant*N*P	2	0.012	0.429
Soil*N*P	1	0.013	0.087
Plant*soil*N*P	2	0.021	0.102

^*a*^ DF, degrees of freedom. Boldface indicates that the *P*-value is less than 0.05.

**Fig 3:**
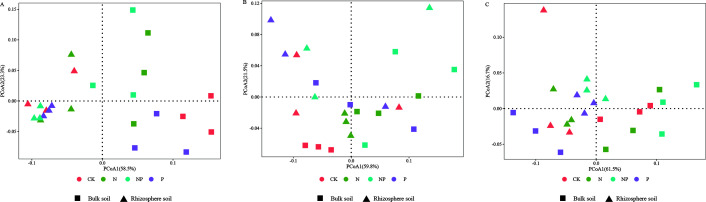
PCoA of the bacterial community of different halophytes at different treatments. (A) *S. salsa*. (B) *P. communis*. (C) *A. sinensis*.

**Fig 4:**
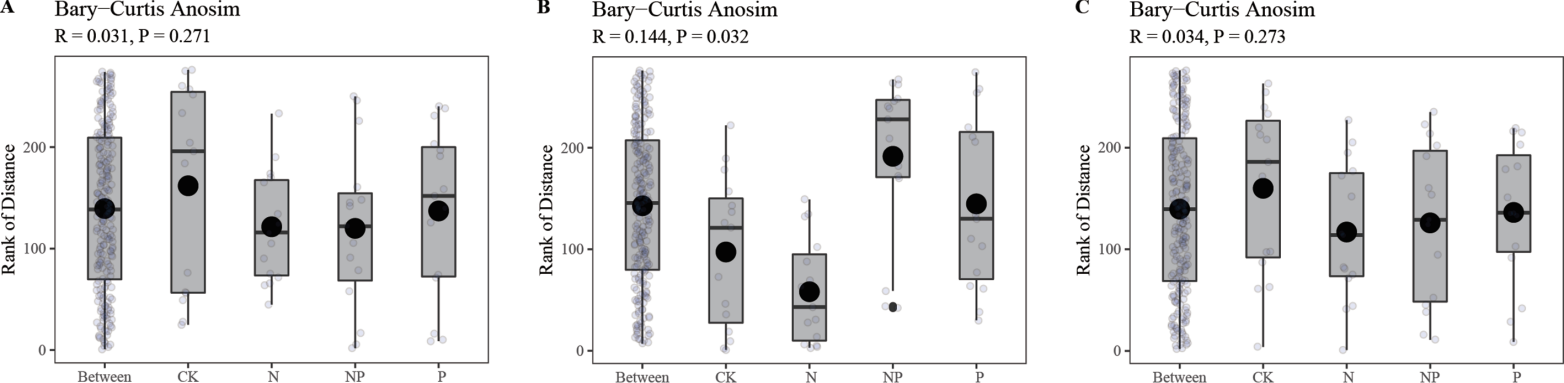
ANOSIM analysis of the bacterial community of different halophytes at different treatments. (A) *S. salsa*. (B) *P. communis*. (C) *A. sinensis*.

The halophyte rhizosphere bacterial community was mainly distributed in the second and the third quadrants (Fig. 3), while the bulk soil bacterial community was mainly distributed in the first and fourth quadrants, indicating the bacterial community structure of bulk and rhizosphere soil was significantly different. ANOSIM test showed that the distance between rhizosphere and bulk soil bacterial community decreased under N and P addition as well as co-addition in both *S. salsa* (Fig. 4A) and *A. sinensis* (Fig. 4B) compared to CK, indicating that N and P addition reduced the difference of bacterial community between rhizosphere and bulk soil. In contrast, in *P. communis*, the difference between bulk and rhizosphere bacterial community structure decreased under N addition treatment and increased under P addition and N and P co-addition treatment (Fig. 4C).

### Soil bacterial composition at the phylum level

**Fig 5:**
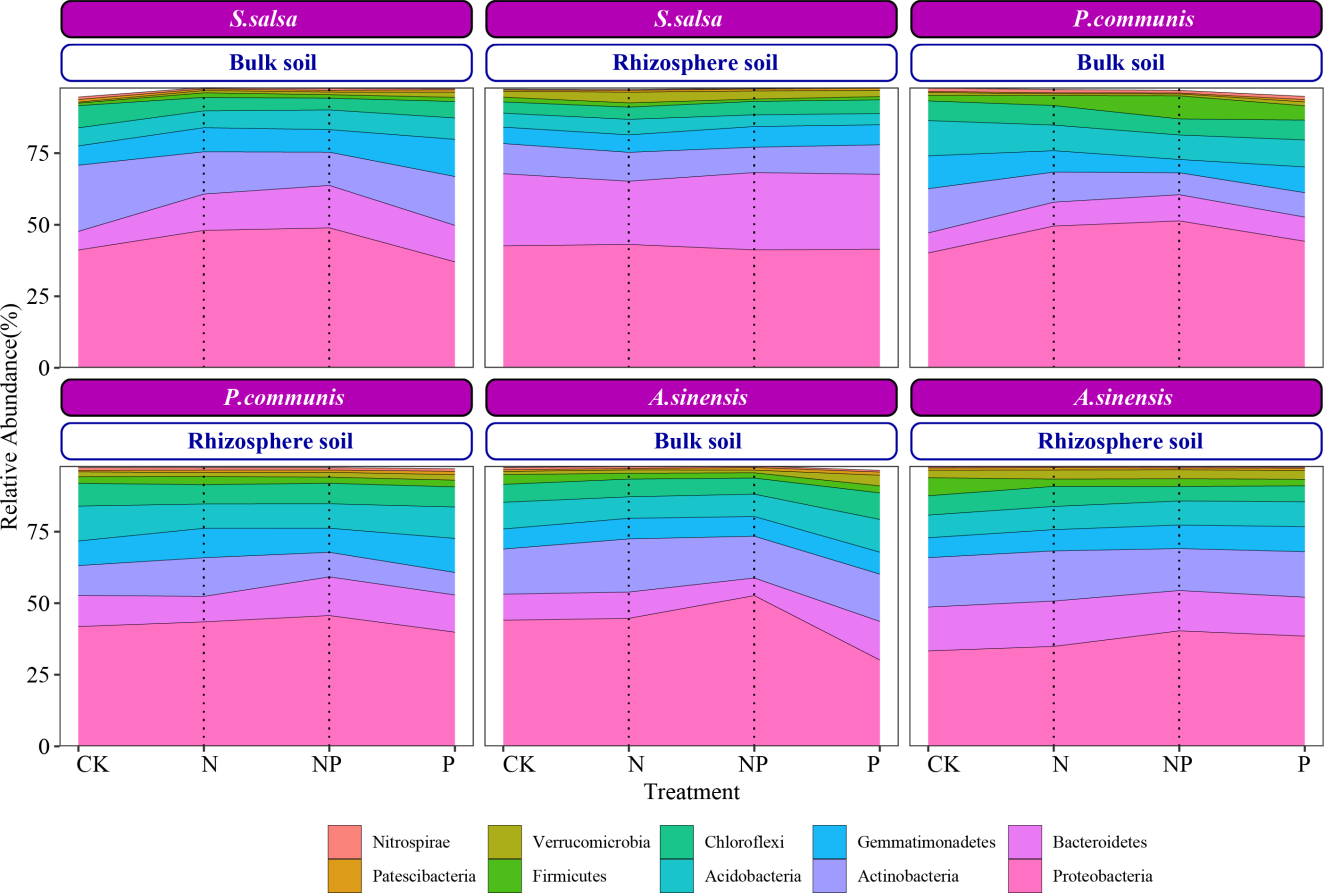
The relative abundance of dominant bacterial phyla of different halophytes at different treatments.

The *Proteobacteria* relative abundance in bulk soil increased under N addition and N and P co-addition and decreased under P addition treatment (Fig. 5; Table S2), which made its relative abundance higher in *S. salsa* and *P. communis* rhizosphere soil than in bulk soil. The *Bacteroidetes* had a higher relative abundance in rhizosphere soil than in bulk soil, while the *Nitrospirae* had a higher relative abundance in bulk soil. Among the plants, the *Bacteroidetes* were more abundant in *S. salsa* than in *P. communis* and *A. sinensis* rhizosphere soil, but the opposite was true for the *Acidobacteria*, *Chloroflexi*, and *Firmicutes*. In *P. communis* and *S. salsa*, the rhizosphere *Bacteroidetes* relative abundance increased under P addition and N and P co-addition treatment. The bulk soil *Bacteroidetes* relative abundance was enhanced under N and P addition as well as co-addition treatment. N and P addition decreased the *S. salsa* and *P. communis* rhizosphere *Acidobacteria* relative abundance.

### Relationship between soil environmental factors and bacterial community α-diversity

**Fig 6:**
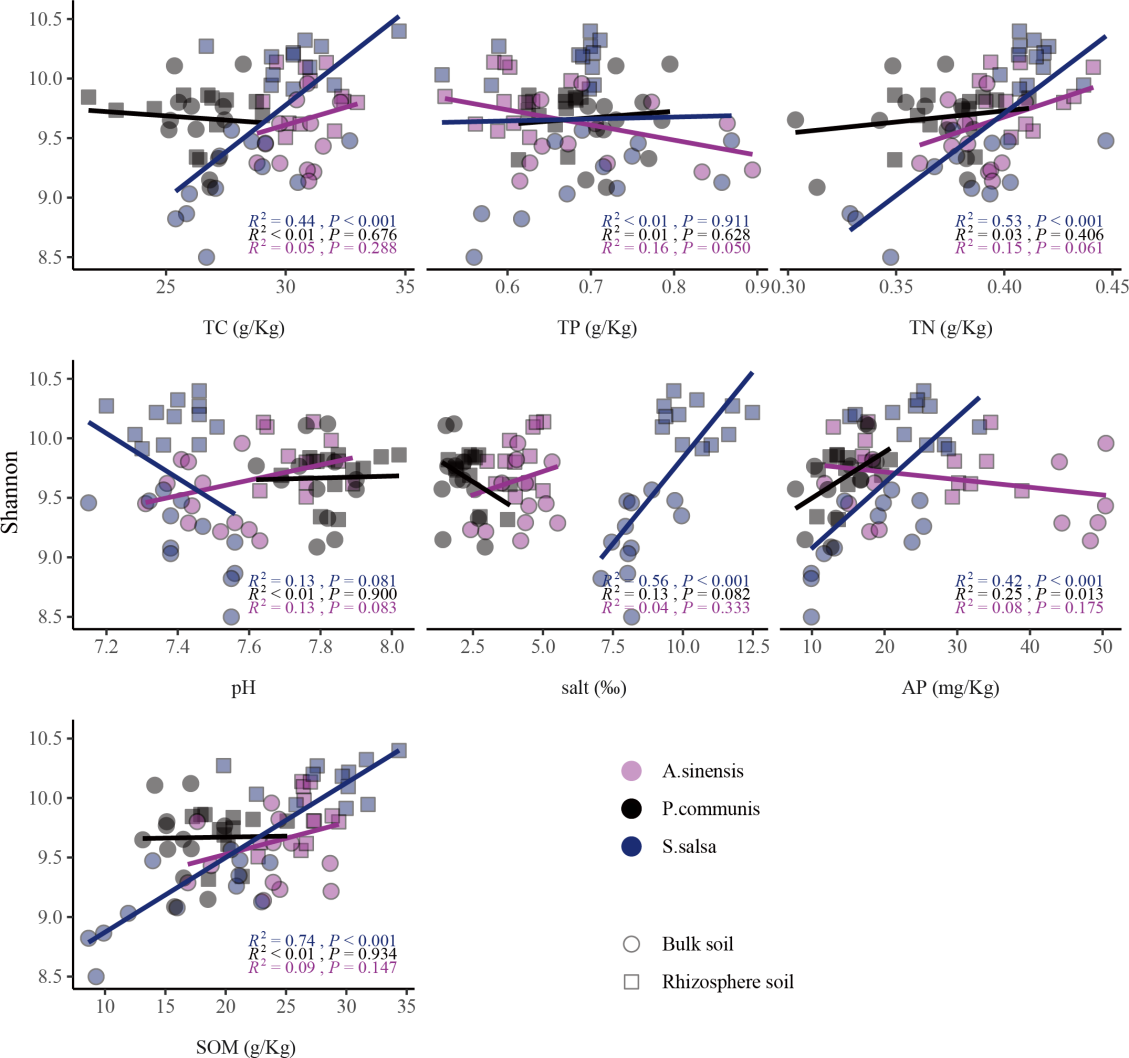
The correlation analysis between the bacterial community Shannon index and the environmental factors.

The *S. salsa* rhizosphere and bulk soil bacterial diversity were most closely related to soil environmental factors (Fig. 6), with SOM (*R*^2^ = 0.74, *P* < 0.001), salt (*R*^2^ = 0.56, *P* < 0.001), TN (*R*^2^ = 0.53, *P* < 0.001), TC (*R*^2^ = 0.44, *P* < 0.001), and AP (*R*^2^ = 0.42, *P* < 0.001) showed a significant positive correlation. *S. salsa* rhizosphere soil salt, carbon, and nutrient content are higher than bulk soil, making the rhizosphere soil diversity higher than bulk soil. In *P. communis*, bacterial diversity was positively correlated with AP content (*R*^2^ = 0.25, *P* = 0.013). Unlike *S. salsa* and *P. communis*, *A. sinensis* rhizosphere and bulk soil bacterial diversity did not correlate well with AP, while it showed a significant negative correlation with TP (*R*^2^ = 0.16, *P* = 0.05).

### Redundancy analysis

**Fig 7:**
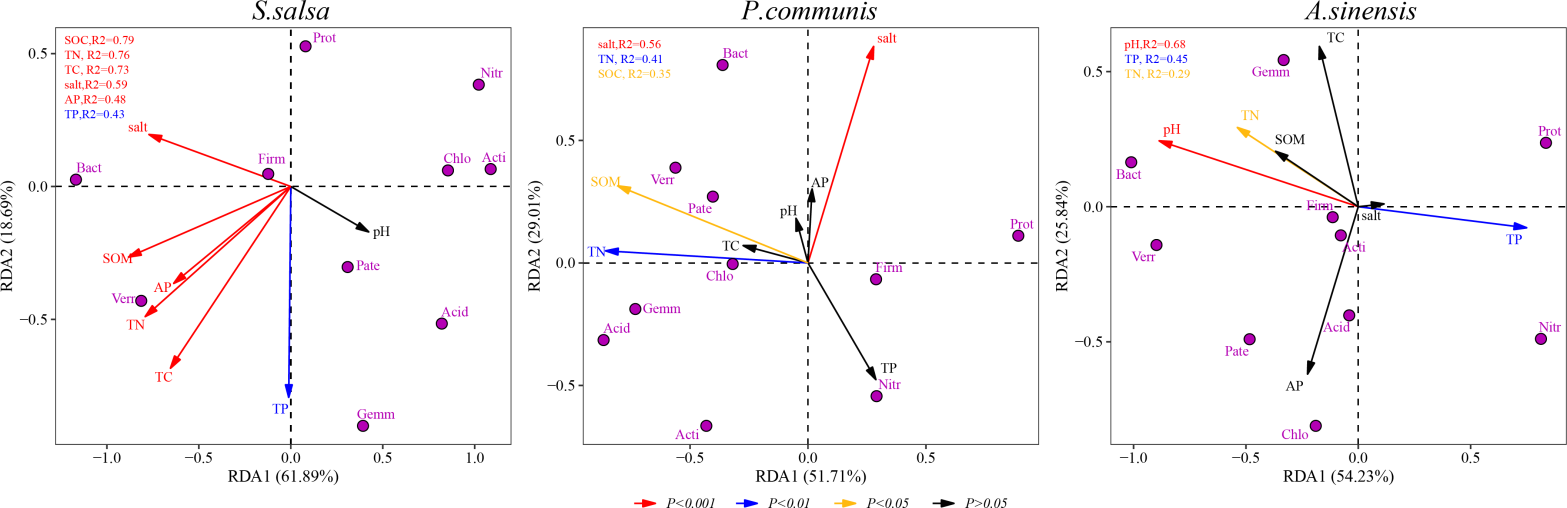
RDA between the bacterial community composition and environmental factors. The red, blue, and yellow lines indicated that the effect of soil environmental factor on bacterial community is significant at 0.001, 0.01, and 0.05, respectively.

RDA and Monte Carlo tests were used to analyze the relationship between soil environmental factors and bacterial community structure (Fig. 7; Table 3). The SOM, TN, salt, AP, TP, TC, and pH content were used as environmental factors, and the top 10 phyla were used as bacterial community fraction. Among the three plant species, the association of bacterial community structure with environmental factors was stronger in *S. salsa* while weaker in *P. communis* and *A. sinensis*. The TN content had a significant effect on bacterial community structure, explaining 76%, 41%, and 29% of variation in *S. salsa*, *P. communis*, and *A. sinensis*, respectively. The key environmental factor driving changes in bacterial community structure were inconsistent among the three plant species. In *S. salsa*, SOM content had the greatest effect on bacterial community composition, explaining 79% of the variation of the bacterial community (*P* < 0.001); in addition, Monte Carlo tests showed that TC, salt, AP, and TP content had significant effects on bacterial community structure. SOM and salt content explained 35% (*P* < 0.05) and 56% (*P* < 0.001) of the variation in *P. communis* bacterial community structure, respectively. pH and TP explained 68% (*P* < 0.001) and 45% (*P* < 0.01) of the variation in community composition in the community structure of *A. sinensis*, respectively. Among the three species, the *Proteobacteria*, *Nitrospirae* had a strong negative correlation with the C content and N content, while the *Bacteroidetes* had a strong positive correlation with it. In *S. salsa* and *P. communis*, salt content was positively correlated with the abundance of *Bacteroidetes* and negatively correlated with the abundance of *Gemmatimonadetes*, *Actinobacteria*, and *Acidobacteria*.

**TABLE 3:** Monte Carlo test of effects of soil physicochemical property on the bacterial community structure of halophytes^*a*^

Property	*S. salsa*		*P. communis*		*A. sinensis*	
	*R*	*P*	*R*	*P*	*R*	*P*
TC	0.732	**0.001**	0.040	0.634	0.212	0.094
TP	0.429	**0.004**	0.138	0.228	0.457	**0.007**
TN	0.765	**0.001**	0.407	**0.002**	0.293	**0.036**
pH	0.190	0.121	0.018	0.839	0.682	**0.001**
Salt	0.591	**0.001**	0.561	**0.001**	0.010	0.892
AP	0.480	**0.001**	0.054	0.565	0.214	0.084
SOM	0.792	**0.001**	0.354	**0.012**	0.139	0.209

^*a*^ The boldface indicates that the *P*-value is less than 0.05.

## DISCUSSION

### Effect of N and P addition on rhizosphere and bulk soil bacterial community diversity

Environmental change can alter the community composition of wetland soil microorganisms, and in turn, changes in wetland soil microorganisms can lead to changes in the overall structure and function of their ecosystems (25). Wetland plants interact with environmental factors to influence soil microbial community and functional diversity (25). Zhao et al. (26) studied the rhizosphere soil bacterial community of three different halophytes in the YRD and found that *P. communis* bacterial diversity and richness were higher than those of *S. salsa* and *A. sinensis*. However, in this study, it was found that the rhizosphere soil bacterial community of *S. salsa* had a high diversity, which was not consistent with the previous study. This may be due to the higher salt and SOM content and lower pH in *S. salsa* rhizosphere soil, all three of which behaved opposite to *P. communis* and *A. sinensis* bulk soil. Higher salt and lower pH did not promote bulk soil bacterial diversity in *S. salsa*; therefore, we suggest that it may be the SOM content that causes the differences in soil bacterial diversity among the three halophytes. There was a strong correlation between soil bacterial diversity and rhizosphere soil SOM of *S. salsa* in this study, which is consistent with the previous study (27). In addition, plants shaped the soil bacterial community through selection and used the deposits to influence the diversity of the soil bacterial community (28). Plant residues are the main source of nutrition for plant rhizosphere soil bacteria (27). The amount and variety of apoplastic material vary greatly among plants, which in turn leads to differences in bacterial community diversity among the three halophytes.

Increasing nutrition with bio-limitation can directly change bacterial community structure or indirectly affect bacterial community structure by changing plant biomass. For example, the nutrient changes the root structure, thus providing different microhabitats for the bacterial community, and can change the bacterial community composition in the form of root secretions and apoplastic inputs (18). Yan et al. (29) found that N addition did not affect bacterial community diversity and richness, while P addition reduced bacterial community diversity. Wang et al. (8) found that N addition reduced bacterial diversity and richness, while P addition did not mitigate the negative effect of N addition on the bacterial community. In this study, we found that three halophyte soil bacterial community diversity responses were incoherent to N and P addition. This may be due to the difference in the nutrient requirement of the plant. Previous studies found that *P.communis* rhizosphere soil N content is usually low, and N-fixing bacteria are abundant. N addition is known to inhibit the growth of N-fixing bacteria (26, 30, 31), which may have broken the positive interaction between the *P. communis* and bacterial community and thus reduced the *P. communis* rhizosphere bacterial diversity. We further analyzed the relative abundance of *alphaproteobacteria* and found that N addition decreased its abundance in *P. communis* (Fig. S2). However, despite the decrease in the relative abundance of *alphaproteobacteria* caused by N addition, the decrease did not reach a significant level.

N addition alleviates nutrient limitation in the growth of *S. salsa* and *A. sinensis* and increases the SOM input to the soil, which can enhance the growth of the bacterial community (7, 32, 33). Previous studies have found that the response of the bacterial community to P addition varied according to the changes in the effectiveness of soil carbon. When soil carbon is high, microbial biomass and activity may be limited by P (8). In this study, the *P. communis* and *A. sinensis* rhizosphere SOM content was lower than *S. salsa*, and P addition promoted the rhizosphere soil bacterial communities diversity of *P. communis* and *A. sinensis* while reducing the rhizosphere soil bacterial diversity of *S. salsa*.

N addition is usually accompanied by soil acidification, leading to a decrease in bacterial community diversity (15, 34). To be mentioned, most of these studies were based on long-term N and P addition experiments, while the present study was a short-term N and P addition. The effect of N and P addition on the bacterial community diversity changed with the plants, which indicated that the effect of plants on the bacterial community was much greater than the short-term N and P addition.

### Effects of N and P addition on the composition of rhizosphere and bulk bacterial community

Soil bacterial community composition was influenced by soil and plants (35). Therefore, we predicted that the bacterial community composition would change after nutrient addition, and the trend of changing rhizosphere bacterial composition might vary among halophytes. PCoA showed that N addition significantly changed the bulk soil community composition of the three halophytes, which is consistent with previous studies (15, 36). However, N addition had no significant effect on the rhizosphere soil bacterial community composition of *S. salsa* and *P. communis*. In general, bacterial communities of plant rhizosphere soil have lower β-diversity and are more stable compared to that of bulk soils (37). In addition, in terms of ecological construction processes, stochastic processes contributed more to the rhizosphere soil bacterial community, while deterministic processes contributed more to the bulk soil (37), which makes rhizosphere bacterial community composition less responsive to environmental change than bulk soil. In *S. salsa* and *A. sinensis*, N and P addition reduced the difference in bacterial community composition between bulk and rhizosphere soil, which may be due to the increase in rhizosphere soil nutrition caused by N and P addition, resulting in a decrease in soil nutrient difference.

Previous studies have found that *Proteobacteria*, *Actinobacteria*, and *Bacteroidetes* belong to eutrophic bacteria groups, and *Chloroflexi* and *Acidobacteria* belong to oligotrophic microbial groups (13, 38, 39). We predicted that nutrient addition would lead to the rapid growth of eutrophic taxa and decrease oligotrophic taxa. In this study, N and P addition reduced the *Acidobacteria* relative abundance in the rhizosphere and bulk soil bacterial community of *P. communis*, which further reflects the oligotrophic survival strategy of the *Acidobacteria*. Unexpectedly, P addition reduced the relative abundance of *Proteobacteria* in this study. This could be because the bacterial growth of the three *S*. *salsa* is N-limited, and P addition exacerbates the N-limitation of bacterial growth. There was more adequate nutrition in the N and P addition treatment, so *Proteobacteria* was the highest.

The rhizosphere soil *Bacteroidetes* contained a high relative abundance, and RDA showed that both salt and SOM content were significantly and positively correlated with *Bacteroidetes*. A previous study found that *Bacteroidetes* have good salt tolerance and resistance, and higher salt level promotes the growth of salt-tolerant bacteria (40). *S. salsa* are salt aggregating plants and can take up salt from the soil through the root system to accumulate in the body without harm, which results in high rhizosphere soil salt content (26, 40). In addition, according to the trophic survival strategy of bacteria, rhizosphere soil is abundant in C sources, which lead to a high relative abundance of the *Bacteroidetes* in rhizosphere soil (41). The addition of N and P enhanced the relative abundance of Bacteroidetes in *S. salsa* and *P. communis* soil, which further confirmed the growth strategy of the *Bacteroidetes*.

In contrast to *Bacteroidetes*, the *Gemmatimonadetes* and *Actinobacteria* have lower salt tolerance or are more susceptible to high salt (26). In this work, both *Gemmatimonadetes* and *Actinobacteria* were negatively correlated with salt content, and the relative abundance of both was less in *S. salsa* rhizosphere soil, which was further corroborated by the previous study. However, the *Actinobacteria* was not relatively more abundant in the rhizosphere soil of *P. communis* and *A. sinensis* due to the lower salt content. However, the *Actinobacteria* was not relatively more abundant in *P. communis* and *A. sinensis* rhizosphere soil due to the lower salt content. This is because *P. communis* is an aquatic plant suitable for survival in a water-rich environment, while the growth of *Actinobacteria* is usually inhibited by water (42). Although *Actinobacteria* belong to eutrophic taxa, they still have a strong competitive advantage in soil with low carbon content, which results in low-SOM content but a high abundance of *Actinobacteria* in *A. sinensis* rhizosphere soil. The *Chloroflexi* had a high abundance in *P. communis* and *A. sinensis* soil, which is consistent with the survival strategy of oligotrophic bacterial taxa. The *P. communis* and *A. sinensis* soil SOM content were lower than that of the *S. salsa*, which promoted the growth and development of *Chloroflexi*, which can use CO_2_ as a carbon source for photosynthesis to obtain energy (43).

### Conclusion

The response of rhizosphere and bulk soil bacterial community α-diversity to N and P addition differed among the three halophytes. N addition increased rhizosphere soil bacterial community α-diversity in *S. salsa* and *A. sinensis*, while decreased that in *P. communis*. P addition decreased rhizosphere bacterial α-diversity in *S. salsa* while increased rhizosphere bacterial α-diversity in *P. communis* and *A. sinensis*. SOM content resulted in the difference between the rhizosphere soil bacterial α-diversity of the three halophytes. TN, SOM, and salt content have strong effects on bacterial community composition. The effect of halophyte on bacterial community composition is greater than that of N and P addition. This study demonstrated that the effects of N and P addition on soil bacterial community were influenced by plant species and root system. The effects of plants on microorganisms should be considered in future studies.

## Data Availability

The data sets presented in this study can be found in NCBI BioProject under accession numbers PRJNA1097356, PRJNA949158, PRJNA935937, and PRJNA925463.

## SUPPLEMENTAL MATERIAL

Supplemental material msphere.00226-24-s0001.docx Fig. S1, Fig. S2, Table S1, and Table S2.

ASM does not own the copyrights to Supplemental Material that may be linked to, or accessed through, an article. The authors have granted ASM a non-exclusive, world-wide license to publish the Supplemental Material files. Please contact the corresponding author directly for reuse.
