# Supplementary material for: Effects of short-term nitrogen and phosphorus addition on soil bacterial community of different halophytes
Source: mSphere. 2024 Apr 29;9(5):e00226-24. doi: 10.1128/msphere.00226-24 (PMC11237384; doi:10.1128/msphere.00226-24)
Supplement: Supplemental material — Fig. S1, Fig. S2, Table S1, and Table S2. [file msphere.00226-24-s0001.docx]

Table S1 PERMANOVA test (per=999) of bacterial community composition. RS, rhizosphere soil. BS, bulk soil.

|  |  | RS | | |  | BS | | |
| --- | --- | --- | --- | --- | --- | --- | --- | --- |
| Plant | Factors | R^2^ | F | Pr(>F) |  | R^2^ | F | Pr(>F) |
| *S.salsa* | N | 0.028 | 0.325 | 0.799 |  | 0.336 | 6.966 | **0.001** |
|  | P | 0.230 | 2.630 | 0.076 |  | 0.170 | 3.528 | **0.025** |
|  | N*P | 0.044 | 0.500 | 0.651 |  | 0.109 | 2.260 | 0.133 |
| *P.communis* | N | 0.089 | 0.940 | 0.404 |  | 0.261 | 3.803 | **0.035** |
|  | P | 0.108 | 1.135 | 0.333 |  | 0.165 | 2.411 | **0.096** |
|  | N*P | 0.042 | 0.444 | 0.698 |  | 0.026 | 0.375 | 0.798 |
| *A.sinensis* | N | 0.022 | 0.285 | 0.869 |  | 0.317 | 7.957 | **0.012** |
|  | P | 0.303 | 3.899 | **0.005** |  | 0.076 | 1.893 | 0.168 |
|  | N*P | 0.052 | 0.670 | 0.59 |  | 0.288 | 7.220 | **0.008** |

Table S2 The relative abundance of top 10 phyla. RS, rhizosphere soil. BS, bulk soil.

| Plant | Soil | Treatment | Nitrospirae | Patescibacteria | Verrucomicrobia | Firmicutes | Chloroflexi | Acidobacteria | Gemmatimonadetes | Actinobacteria | Bacteroidetes | Proteobacteria |
| --- | --- | --- | --- | --- | --- | --- | --- | --- | --- | --- | --- | --- |
| *S.salsa* | BS | CK | 0.89±0.18 | 0.9±0.25 | 0.36±0.02 | 0.88±0.2 | 7.74±1.67 | 6.32±0.04 | 6.75±0.52 | 23.12±3 | 6.5±1.08 | 41.18±2.53 |
|  |  | N | 0.49±0.13 | 0.58±0.08 | 0.78±0.14 | 1.61±0.11 | 4.7±0.47 | 5.88±0.59 | 8.42±1.48 | 14.78±3.66 | 12.68±3.15 | 48.03±6.96 |
|  |  | NP | 0.64±0.1 | 0.6±0.47 | 0.91±0.41 | 1.22±0.38 | 4.1±0.29 | 6.88±0.69 | 7.88±2.72 | 11.62±3.26 | 14.82±3.53 | 48.92±7.82 |
|  |  | P | 0.39±0.21 | 0.83±0.21 | 1.81±0.85 | 1.45±0.51 | 5.75±1.28 | 7.47±1.59 | 12.99±3.53 | 17.07±1.97 | 12.75±2.79 | 37.04±3.93 |
|  | RS | CK | 0.25±0.07 | 0.44±0.1 | 2.05±0.54 | 1.65±1.28 | 3.94±0.38 | 4.88±1.12 | 5.74±0.58 | 10.53±0.48 | 25.2±3.67 | 42.63±3.23 |
|  |  | N | 0.24±0.02 | 0.67±0.19 | 3.74±2.19 | 1.48±1 | 4.27±0.72 | 5.42±0.84 | 6.11±0.87 | 10.08±1.55 | 22.09±2.89 | 43.13±4.95 |
|  |  | NP | 0.21±0.04 | 0.69±0.12 | 2.83±0.59 | 0.8±0.23 | 4.69±0.08 | 4.09±0.22 | 7.23±0.64 | 8.86±0.13 | 27.02±0.53 | 41.22±1.02 |
|  |  | P | 0.23±0.01 | 0.74±0.05 | 2.19±0.63 | 1.13±0.9 | 4.79±0.16 | 3.93±0.31 | 6.98±0.62 | 10.33±0.99 | 26.19±0.28 | 41.42±0.69 |
| *P.comm unis* | BS | CK | 1.15±0.02 | 0.39±0.03 | 0.98±0.17 | 1.95±0.57 | 6.88±0.04 | 12.31±0.82 | 11.49±0.14 | 15.45±0.59 | 7.01±0.67 | 40.13±1.71 |
|  |  | N | 1.14±0.11 | 0.34±0.07 | 0.61±0.16 | 3.49±0.07 | 6.84±1.35 | 8.96±0.8 | 7.48±2.59 | 10.5±1.68 | 8.35±1.36 | 49.53±5.09 |
|  |  | NP | 0.91±0.27 | 0.32±0.04 | 0.66±0.26 | 8.09±7.56 | 5.61±1.94 | 8.56±2.5 | 4.64±0.74 | 7.67±2.26 | 9.15±4.19 | 51.35±9.99 |
|  |  | P | 1.15±0.28 | 0.79±0.45 | 1.38±0.9 | 4.97±2.97 | 6.93±0.66 | 9.44±2.16 | 8.99±2.86 | 8.54±0.9 | 8.45±2.73 | 44.23±5.73 |
|  | RS | CK | 0.97±0.51 | 0.53±0.23 | 1.68±0.56 | 2.35±0.52 | 7.94±1.72 | 12.19±1.43 | 8.6±2.76 | 10.41±1.48 | 10.89±4.77 | 41.85±9.8 |
|  |  | N | 0.89±0.24 | 0.84±0.15 | 1.35±0.39 | 2.87±1.44 | 6.77±0.98 | 8.5±0.16 | 10.28±2.14 | 13.51±1.57 | 8.92±0.23 | 43.47±0.42 |
|  |  | NP | 0.85±0.23 | 0.8±0.47 | 1.7±1.17 | 2.13±0.46 | 7.15±3.31 | 8.55±2.34 | 8.41±3.43 | 8.59±1.79 | 13.51±2.78 | 45.68±11.52 |
|  |  | P | 0.89±0.28 | 1.17±0.63 | 1.92±0.72 | 2.35±0.23 | 7.02±2.42 | 10.98±2.73 | 11.92±5.23 | 7.84±3.06 | 13.01±3.9 | 39.87±7.95 |
| *A.sinensis* | BS | CK | 0.77±0.22 | 0.6±0.09 | 1.1±0.02 | 3.4±1.61 | 6.26±0.36 | 9.33±1.05 | 7.01±0.96 | 15.75±2.49 | 9.12±2.54 | 44.05±3.81 |
|  |  | N | 0.74±0.19 | 0.49±0.05 | 1.15±0.21 | 2.05±0.64 | 6.19±1.16 | 7.51±0.8 | 7.21±0.01 | 18.57±3.35 | 9.19±0.45 | 44.7±5.65 |
|  |  | NP | 0.52±0.09 | 0.91±0.29 | 0.92±0.12 | 1.84±0.66 | 5.62±1.41 | 7.81±0.72 | 6.89±0.49 | 14.6±2.62 | 6.15±1.31 | 52.66±4.27 |
|  |  | P | 0.61±0.27 | 1.1±0.23 | 3.73±1.71 | 2.46±0.93 | 9.27±1.89 | 11.43±3.98 | 7.68±2.77 | 16.52±5 | 13.49±2.61 | 30.15±3.05 |
|  | RS | CK | 0.4±0.08 | 0.78±0.25 | 2.62±0.94 | 6.31±7.86 | 6.75±1.59 | 7.92±1.29 | 6.95±1.17 | 17.28±3.45 | 15.3±2.19 | 33.34±1.83 |
|  |  | N | 0.43±0.05 | 0.87±0.22 | 3.17±0.49 | 2.54±1.38 | 7±0.52 | 8.1±0.94 | 7.43±0.71 | 17.53±0.43 | 15.86±3.06 | 34.9±1.55 |
|  |  | NP | 0.42±0.09 | 0.66±0.13 | 3.11±1.17 | 2.69±0.49 | 5.07±0.23 | 8.4±0.33 | 8.24±0.41 | 14.65±1.49 | 14.09±1.26 | 40.35±1.31 |
|  |  | P | 0.36±0.02 | 0.8±0.06 | 3.07±0.36 | 2.31±1.28 | 5.54±0.52 | 8.71±0.86 | 8.75±1.2 | 15.9±1.24 | 13.6±0.88 | 38.5±1.4 |


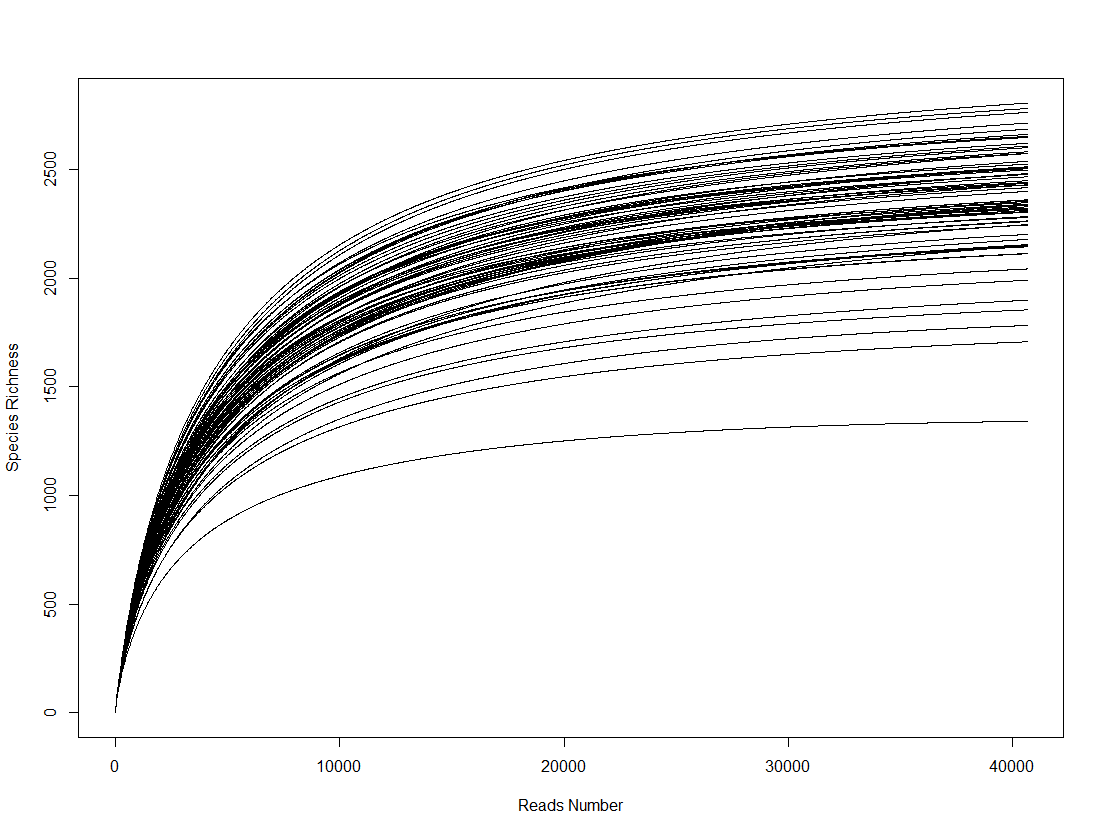


Figure S1 Species accumulation curves


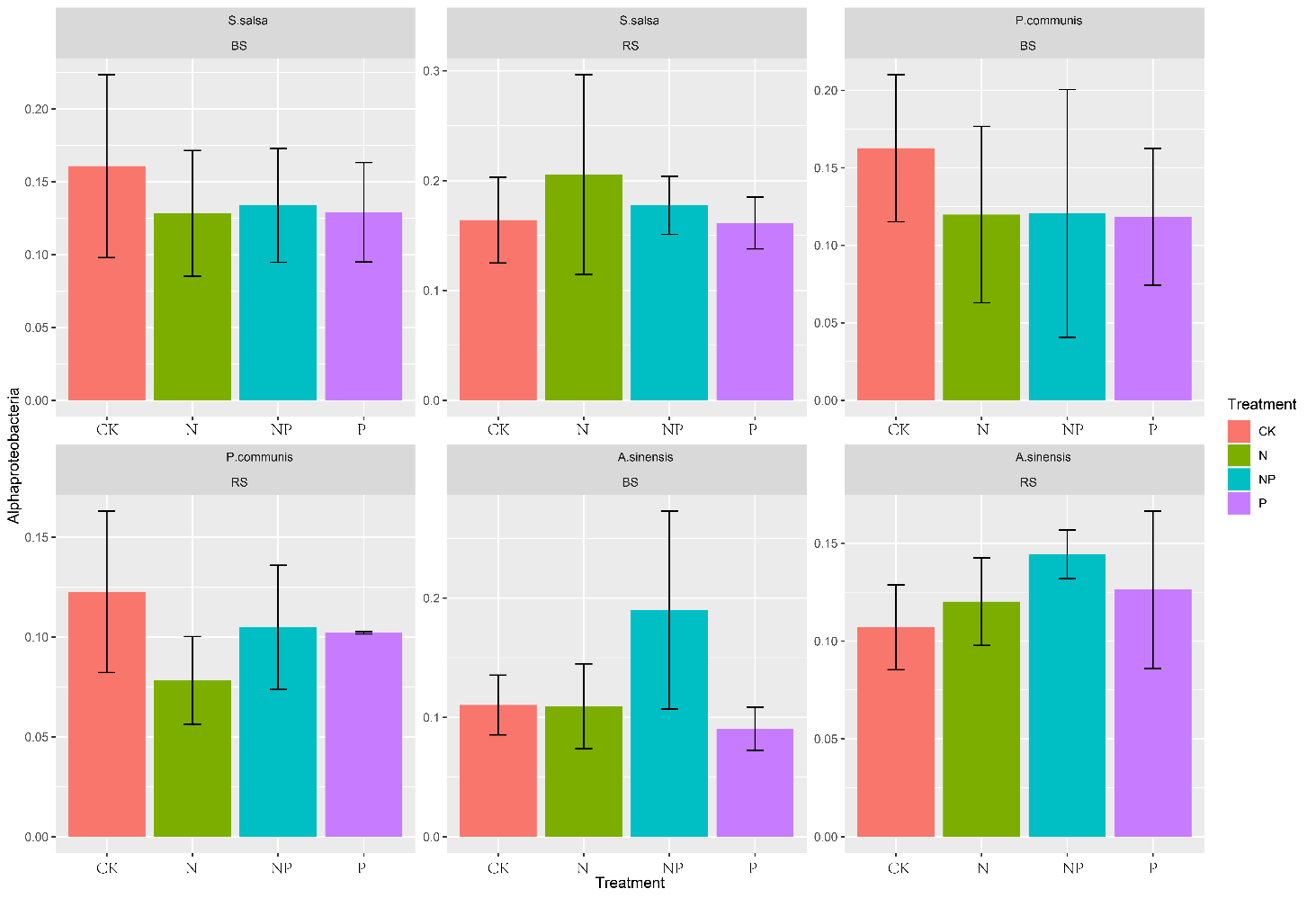


Figure S2 The relative abundance of alpha-proteobacteria. BS, bulk soil. RS, rhizosphere soil.
